# Different Chemical Structures and Physiological/Pathological Roles of Cyclooxygenases

**DOI:** 10.5041/RMMJ.10426

**Published:** 2021-01-19

**Authors:** Yalcin Faki, Ayse Er

**Affiliations:** Department of Pharmacology and Toxicology, Faculty of Veterinary Medicine, University of Selcuk, Konya, Turkey

**Keywords:** Brain, cyclooxygenase, digestive system, heart, kidney, pathologic status, physiologic status, platelets

## Abstract

This review describes cyclooxygenase (COX), which synthesizes prostanoids that play an important role in living things. The authors conducted a national and international literature review on the subject. The COX enzyme uses arachidonic acid to form prostanoids, which play a role in several physiological and pathological conditions. This enzyme has different isoforms, mainly COX-1 and COX-2. The constitutive isoform is COX-1, while COX-2 is the inducible isoform. Both are expressed in different tissues and at different levels, but they may also coexist within the same tissue. Both isoforms show essentially the same mode of action, but their substrates and inhibitors may differ. The COX-1 isoform, which plays a role in the continuation of physiological events, has an increased expression level in various carcinomas, and the COX-2 isoform, which is increased in inflammatory conditions, is typically expressed at low physiological levels in some tissues such as the brain, kidney, and uterus. In addition to investigating the efficacies of the COX-1 and COX-2 isoforms, the discovery of potential new COX enzymes and their effect continues. This review also looks at the roles of the COX enzyme in certain physiological and pathological conditions.

## INTRODUCTION

Cyclooxygenase (COX) is a homodimer and mono-topic membrane protein.^1^ The COX enzyme converts arachidonic acid to prostanoids. It was first discovered in 1971 in a study that demonstrated the mechanism of action of non-steroidal anti-inflammatory drugs (NSAIDs).^2^ The COX enzyme (also called prostaglandin [PG] G/H synthase or prostaglandin endoperoxidase H synthase) catalyzes two separate reactions: the cyclization of arachidonic acid to form PGG2 and the hydroperoxidation for PGH2 generation from PGG2.^3^,^4^ When it was first discovered, COX was reported to have a uniform structure, but later studies revealed two isoforms, COX-1 (PGHS-1) and COX-2 (PGHS-2).^3^,^5^ In 1989, molecular biologists identified an early gene similar to the COX-1 isoform.^6^ In 1991, a second COX enzyme was reported, called COX-2, which is separate from the COX-1 isoform.^7^ The discovery of COX-2 was a major advance in COX enzyme research.^8^ Although there is no functional difference between the COX-1 and COX-2 isoforms, there are differences in their chemical structures, intracellular positions, and biological functions.^9^,^10^

The COX-1 and COX-2 isoforms are membrane-bound proteins that are found primarily in the endoplasmic reticulum (ER) after synthesis and transport.^10^ The COX-1 gene is located on chromosome 9, and the COX-2 gene is located on chromosome 1.^11^ The human COX-2 gene (8.3 kb) is smaller than the COX-1 gene (22 kb), and COX-1 does not have a TATA box. Unlike COX-1, the COX-2 gene is characterized by a TATA box. The COX-1 gene is compatible with fast transcription and mRNA processing so as to process a stable message that is continuously copied. In contrast, the features of the COX-2 gene, although not always present, bear the characteristics of a highly regulated and upregulated “early” gene during inflammation or pathological processes.^10^ The gene products are also different; the mRNA for the inducible enzyme is approximately 4.5 kb, and the mRNA of the constitutive enzyme is 2.8 kb^9^; however, COX-1 and COX-2 have similar molecular weights (70 and 72 kDa, respectively) (Table 1).^13^ The COX-1 and COX-2 signal peptides have 24–26 and 17 amino acids, respectively, both of which are recognized by the signal recognition particle of the ER.^14^ In brief, the COX-2 and COX-1 genes are arranged by two independent and completely different systems, although the enzymatic reaction catalyzed by both isoforms is identical.^15^

**Table 1:** Comparison of the COX-1 and COX-2 Isoforms.

Parameter	COX-1 Isoform	COX-2 Isoform
Style of expression	Constitutive	Inducible

Location	Chromosome 9	Chromosome 1

Relative size of active site	Smaller	Larger

Size of gene	22 kb	8.3 kb

TATA box	-	+

Size of mRNA	2.8 kb	4.5 kb

Molecular weight	70 kDa	72 kDa
Number of amino acids	576*	581†

* 602 amino acids have also been reported.^12^

† 604 amino acids have also been reported.^12^

The amino acid sequences of the COX-2 and COX-1 isoforms share approximately 60%–80% similarity.^12^,^16^ The COX-1 and COX-2 isoforms have been reported to have 576 amino acids^5^ or 602 amino acids^12^ and 581 amino acids^5^ or 604 amino acids,^12^ respectively. The most important differences are in the amino acid sequences at the N (amino) and C (carboxyl) terminals.^10^,^12^ At the N-terminus of the COX-1 isoform, there is a 17-amino-acid sequence not found in the COX-2 isoform. In contrast, the C-terminus of the COX-2 isoform has an 18-amino-acid sequence not found in the COX-1 isoform.^8^,^9^

**Figure 1:**
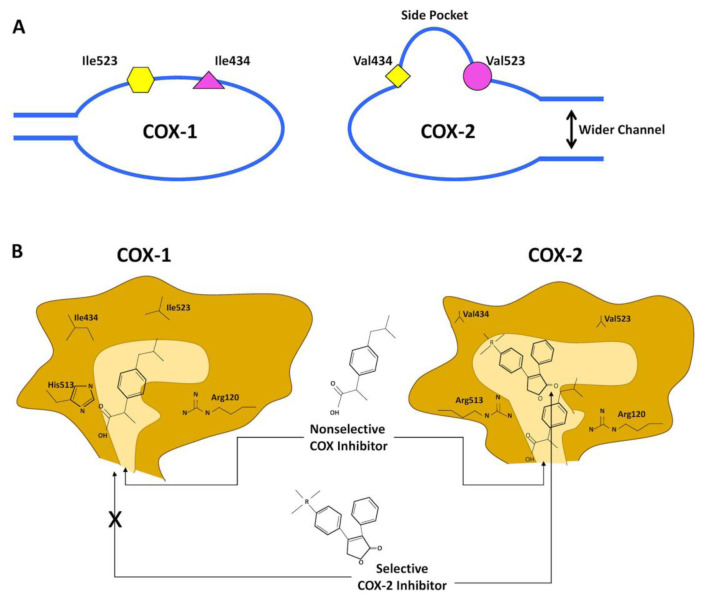
Schematic Depiction of the Structural Differences between the Substrate-binding Channels of Cyclooxygenase (COX)-1 and COX-2. Despite overall similarities, COX-1 and COX-2 show small differences in amino acid composition at the active sites. **A:** COX-1 has isoleucine (Ile) 523 (yellow hexagon) and 434 (pink triangle), while COX-2 has valine (Val) 523 (Val523, pink circle) and 434 (yellow square). This substitution creates a side pocket of the active binding site of COX-2, not present in COX-1. In addition, the channel of the active site in COX-2 is larger than in COX-1. Note that the non-selective COX inhibitor has access to the binding channels of both isoforms. **B:** The more voluminous residues (Ile523, Ile434 and Histidine (His)513) in COX-1 make a narrower entrance channel and obstruct access of the bulky side chains of the selective COX-2 inhibitor. The COX-2 side pocket allows specific binding of the selective COX-2 inhibitor side extension. Arg, Arginine. Based on Grosser et al.^18^

Despite general similarities, COX-1 and COX-2 show differences in their amino acid composition at the active site.^10^ In the COX-2 isoform, there are significant amino acid differences that lead to a larger “side pocket” for substrate access.^17^ In its active region, the presence of valine in COX-2, instead of the isoleucine in COX-1, at positions 434 and 523, makes this region of COX-2 larger than that of COX-1 (Figure 1A).^10^,^19^ As a result, the COX-2 isoform has a wider and more flexible substrate channel than the COX-1 isoform, and the inhibitory binding region in COX-2 is 17%–25% larger than in COX-1 (Figure 1B).^10^,^13^

Histofluorescence staining techniques and confocal fluorescence imaging microscopy experiments have shown that the COX enzyme is found in both the ER and in the nuclear membrane. The COX-1 isoform is localized to the ER, whereas COX-2 is localized to the nuclear membrane and ER. However, the COX-2 isoform is found to be two times more abundant in the nuclear membrane than in the ER.^20^ It has been reported that this selective localization may be caused by a different sequence at the C-terminus.^9^,^10^ The reason that COX enzymes are associated with several different membrane systems is that prostanoid synthesis at different subcellular sites can be achieved by different stimuli.^8^

## COX-1 AND COX-2 IN PHYSIOLOGICAL AND PATHOLOGICAL CONDITIONS

**Figure 2:**
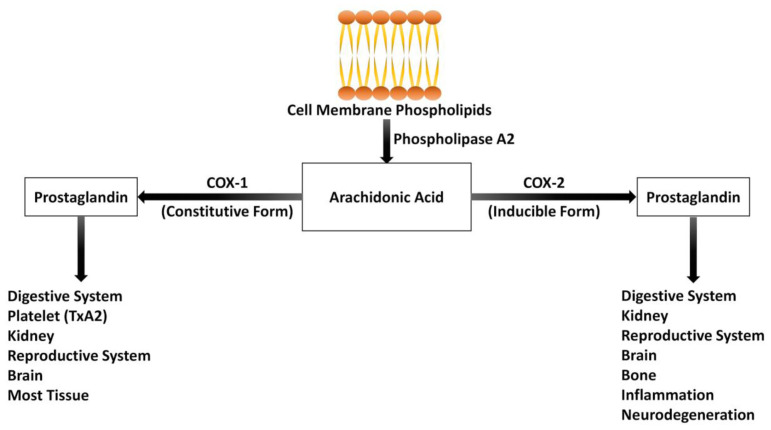
Enzymatic Pathway of Prostaglandin (PG) Formation from Arachidonic Acid and Physiological and Pathological Functions of Cyclooxygenase (COX)-1 and COX-2. Arachidonic acid is released from membrane phospholipids by phospholipase A2, which is activated by various stimuli (inflammatory, physical, chemical, and mitogenic). Cyclooxygenase-1 is constitutively expressed in tissues and produces prostaglandins which are involved in physiological processes. For example, prostacyclin made by the stomach mucosa protects it from damage by gastric acid, thromboxane A2 (TxA2) made by platelets promotes the clotting of blood when required, and PGE2 made in kidney cells is involved in kidney function. Cyclooxygenase-2 is inducibly expressed, but some constitutive COX-2 is also expressed, particularly in the brain. Cyclooxygenase-2 is mainly induced in inflammation and by mitogens and growth factors. It is induced in macrophages and other inflammatory cells by bacterial lipopolysaccharide and cytokines. It forms mainly PGE2, which with other inflammatory mediators causes pain and fever.

The COX enzymes play roles in pathological events as well as in maintaining many physiological functions in living organisms. While COX-1 is continuously synthesized and is involved in the maintenance of physiological events, COX-2 is inducible and plays a role in inflammation.^9^,^15^ Although there are differences between the two isoforms, they use the same endogenous substrate (arachidonic acid) and form the same product via the same catalytic mechanism. The most important differences between them appear in their pathophysiological functions (Figure 2).^9^

Phospholipase A2 hydrolyses membrane phospholipids and releases a 20-carbon polyunsaturated arachidonic acid.^21^ Arachidonic acid is the major prostanoid precursor. Prostanoids are derived from polyunsaturated fatty acids via the COX pathway. In addition, they are members of a large group of hormonally active compounds and act locally, in the vicinity of the region of their production.^3^,^22^

When the cellular concentrations of arachidonic acid are low, metabolism by COX-2 predominates, while COX-1 reactions predominate at high arachidonic acid concentrations.^13^ In one study, COX-2 expression did not change low concentrations of arachidonic acid, whereas PGE, an indicator of inflammation, was significantly increased. Therefore, it has been reported that only high doses of fatty acids can alter COX-2 gene expression and that low doses alter eicosanoid production in other ways.^23^

Despite the structural identity, there are obvious biochemical differences in inhibitor and substrate selectivity. For instance, the COX-2 isoform accepts a wider range of fatty acids as substrates than COX-1; while both isoforms can use dihomo-γ-linolenate and arachidonic acid equally, COX-2 oxygenates other fatty acid substrates (γ-linolenic acid, eicosapentaenoic acid, α-linolenic acid, and linoleic acid) more efficiently than COX-1. In addition, another important difference between COX-1 and COX-2 is their ability to use different substrate pools.^9^,^15^ The COX-1 isoform uses extracellular arachidonic acid, while the COX-2 isoform uses intracellular arachidonic acid.^24^

The most striking distinctions between the COX-1 and COX-2 isoforms are their tissue distributions and the differential regulation of their expression.^8^ The COX-1 isoform is constitutively expressed in almost all tissues (gastrointestinal tract, kidneys, vascular smooth muscle, and platelets).^8^,^17^ The COX-1 isoform likely participates in the housekeeping functions of prostaglandins, such as the cytoprotective effects in the integrity of platelet function, the gastric mucosa, and the maintenance of renal perfusion. There is little information about the elements involved in the regulation of COX-1 gene expression, but it has been reported that COX-1 can be co-induced with COX-2 in inflammatory joint fluid and in carcinoma.^8^,^15^,^17^,^25^ In many tissues, COX-2 cannot be detected, but its expression can be induced by various stimuli due to inflammation in monocytes or mast cells. When induced, protein levels increase within a few hours and then decrease.^8^,^9^,^10^ Therefore, the COX-2 isoform is often called an inducible COX isoform, because it is rapidly expressed in response to growth factors and pro-inflammatory cytokines, as are other immediate early genes.^8^ This demonstrates the role of the COX-2 isoform in both inflammation and in the control of cell growth. Initial studies have shown that COX-2 is an inducible enzyme in cases of inflammation, but more recent studies have also shown that it is expressed in organs such as the kidney and brain.^8^,^9^ In addition, the overexpression of COX-2 has been reported in neurodegenerative disorders like Parkinson’s disease, in chronic inflammatory diseases like rheumatoid arthritis, and in various cancers.^26^

## FUNCTIONAL IMPORTANCE OF COX-1 AND COX-2 ISOFORMS IN VARIOUS ORGANS AND DISEASE STATES

### Digestive System

**Figure 3:**
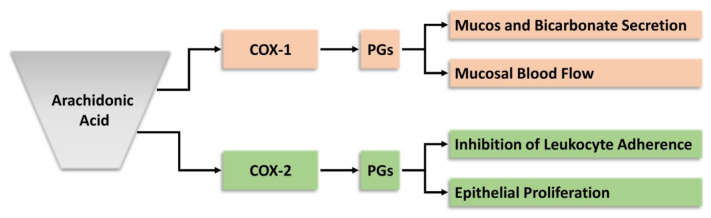
COX-1 and COX-2 in Gastrointestinal Defense. Prostaglandins (PGs) derived from the COX-1/COX-2 isoforms affect different aspects of mucosal defense. Cyclooxygenase-1 produces the PGs that regulate epithelial secretion of mucus and bicarbonate and mucosal blood flow, while COX-2 produces PGs that affect endothelial-leukocyte adherence and epithelial proliferation.

Cyclooxygenase-1 and low levels of COX-2 contribute to gastric mucosal defense, and the isoforms affect different components of this defense.^27^,^28^ Prostaglandins produced by COX-1 regulate the mucosal blood flow and epithelial secretion of mucus and bicarbonate, while PGs produced by COX-2 affect epithelial proliferation and endothelial–leukocyte adherence (Figure 3).^28^

When the COX-2 isoform is inhibited, the absence or underdevelopment of lesions in the stomach indicates that COX-1 is the mediator of gastric mucosal defense under normal conditions.^28^,^29^ Surprisingly, it has been reported that gastric lesions do not develop in mice with COX-1 gene knockout; they experience less gastric damage, bleeding, and ulceration than wild-type mice when administered with an NSAID.^30^ Prostaglandins produced by COX-1 tonically suppress COX-2 activity in the gastrointestinal tract, and the inhibition of COX-1 upregulates COX-2 expression.^28^,^31^ In rat intestine, COX-2 expression might increase as a result of the selective inhibition of COX-1. Recent studies have shown that COX-2 plays a role in modulating resistance to lumen irritants when other factors of mucosal defense are genetically or pharmacologically depressed. For example, when nitric oxide (an important mediator of many components of mucosal defense) synthesis is inhibited, selectively inhibiting the COX-2 enzyme leads to stomach damage. Ischemia-reperfusion causes a marked increase in COX-2 expression in the stomach, and the pre-ischemia inhibition of COX-2 was reported to result in further deterioration of the gastric damage. In another study, peritonitis developed in COX-2 knockout mice, possibly due to impairment of the intestinal barrier function.^28^ In addition, COX-2 null mice are more susceptible to colon damage.^29^ The COX-2 isoform increases rapidly when mucosal damage occurs or when the COX-1 isoform is inhibited. In these cases, suppressing the activity of the COX-2 isoform results in increased mucosal damage and delayed repair.^28^

### Kidney

Prostaglandins have important physiological roles in the modulation of glomerular hemodynamics, the reabsorption of sodium/water, and the regulation of renin secretion.^15^ Both isoforms (but mainly COX-1) are found in the kidney.^8^ However, the relevant kidney data are not clear, and the COX-1 and -2 distribution differs according to the organism.^10^ In this context, COX-1 is preferentially expressed in the renal vessels and in the papillary and medullary collecting ducts in monkeys, humans, rabbits, dogs, and rats, whereas COX-2 expression is limited in epithelial cells of the thick ascending limb, the macula densa adjacent to the juxtaglomerular apparatus, and medullary interstitial cells of rabbits, rats, and dogs. The expression of COX-2 in adult human kidney occurs in endothelial and smooth muscle cells of the arteries/veins and intra-glomerularly in podocytes. It was not found in macula densa of control kidneys, whereas slight–moderate COX-2 expression was found in the macula densa of patients (46%) with diabetic nephropathy, patients (46%) with hypertension, and patients (30%) with congestive heart failure. Also, COX-2 was determined in patients with Bartter-like syndrome. Additionally, COX-2 expression in adult rat was not detected in the arterioles, glomeruli, and cortical/medullary collecting ducts.^8^,^32^–^34^

In the kidneys, COX-2 expression in the macula densa depends on the lumenal salt concentration. In rats, COX-2 expression was found to be increased in the macula densa when the salt intake was restricted (at least 3 weeks).^32^ Cyclooxygenase-2 activates the renin–angiotensin system, while the increased activity of the renin–angiotensin system inhibits the COX-2 isoform.^35^ Recently, COX-2 was found to be detectable in the macula densa of elderly humans (>60 years of age). It was reported that COX-2 expression in elderly subjects may cause a secondary reduction in basal renin production associated with aging.^36^ Cyclooxygenase-2 has major importance in healthy volunteers, and rofecoxib, a COX-2 inhibitor, results in blockage of renin and aldosterone secretion.^37^ Celecoxib is better tolerated in elderly patients, as the risk of acute kidney injury is lower in celecoxib than in rofecoxib.^38^,^39^

Although COX enzyme does not play a dominant physiological role in the kidneys of healthy individuals, the COX-1 isoform is primarily involved in the control of renal hemodynamics and the glomerular filtration rate, whereas the COX-2 isoform is primarily involved in salt and water excretion. The COX-2 isoform is regulated in response to intravascular volume. Thus, the inhibition of one or both of these enzymes may have different effects on renal function.^9^,^35^

The COX-2-selective inhibitors and NSAIDs may impair the systemic and renal vasodilatory effects of prostacyclin. Loss of this vasodilation effect may lead to increases in systemic vascular resistance and then increases in mean arterial pressure.^40^ Rofecoxib (not celecoxib or naproxen) caused a significant increase in the systolic blood pressure of osteoarthritis patients.^41^ Rofecoxib but not celecoxib increased congestive heart failure.^42^ Non-steroidal anti-inflammatory drugs (COX-2-selective and non-selective) can be used carefully in arthritis patients with stable cardiovascular disorders and hypertension (not including congestive heart failure and moderate–severe kidney dysfunction).^40^ The COX-2 isoform is expressed in adult and fetal kidney and is required for normal renal development. It has been reported that renal development is impaired in COX-2-deficient mice.^43^,^44^ After mother’s use of nimesulide (selective COX-2 inhibitor NSAID) as tocolytic, the lack of effect of nimesulide on fetal renal and ductal function indicates that fetal prostaglandin synthesis is mainly mediated by the COX-1 isoform.^45^ However, some studies reported neonatal irreversible end-stage renal failure or severe oligohydramnios after use of nimesulide as a tocolytic.^46^,^47^

While the COX-1 protein levels did not change in the kidneys of cirrhotic rats, COX-2 protein expression was increased in the corticomedullary region in these animals. Although urinary PGE2 excretion was equally reduced by both selective COX-1 and COX-2 inhibitors in these animals, urinary sodium excretion, the glomerular filtration rate, and renal plasma flow significantly decreased, and the diuretic and natriuretic responses to furosemide were markedly impaired only when COX-1 was selectively inhibited. The selective inhibition of both isoforms in cirrhotic rats did not affect renal water metabolism. These results indicate that preservation of renal function in cirrhotic rats is dependent on the COX-1 isoform despite the abundant expression of renal COX-2 protein.^48^

### Reproductive System

The COX-1 and COX-2 isoforms are present in the reproductive system.^36^,^49^,^50^ Although both isoforms contribute to the formation of the corpus luteum, COX-1 has been reported to have a greater role than COX-2 in corpus luteum function.^51^

Female reproductive functions such as ovulation, implantation, and decidualization are dependent on the COX-2 isoform.^36^ Multiple disorders of reproductive function relating to ovulation, fertilization, implantation, and decidualization are seen in COX-2-null mice.^10^,^15^ The induction of COX-2 is necessary for the successful ruptures of the follicles and likely directly mediates the activation or generation of proteolytic enzymes necessary for ovulation.^15^ Defective ovulation and failure of fertilization have been reported in female mice lacking the COX-2 isoform. Female mice lacking COX-2 produce fewer eggs, are not fertile, and the development of eggs is abnormal.^52^ Furthermore, the survival of several offspring born from homozygous COX-1-knockout mice highlights the importance of the COX-1 isoform.^30^

The COX-1 and COX-2 genes are differentially regulated in the peri-implantation mouse uterus. Cyclooxygenase-1 is involved in decidualization and/or ongoing localized endometrial vascular permeability, while COX-2 is involved in angiogenesis for placenta formation.^53^ During implantation in humans, the COX-1 isoform is mainly found in the glandular and luminal epithelium, and the COX-2 isoform is primarily found in the luminal epithelium and perivascular cells.^54^ Initially, COX-2 plays a role in mediating the uterine decidual response, but it is not necessary to sustain embryo development and decidual growth during the remainder of pregnancy.^10^

Both COX-1 and COX-2 have been identified in the amnion, chorion, and decidua.^55^ Increased COX-2 expression in the amnion and chorion–decidua was associated with delivery, whereas COX-1 expression was unchanged. In the amnion, COX-1 expression is approximately 100 times lower than COX-2 expression and does not change with delivery.^49^,^56^ Expression of COX-2 begins to increase from early pregnancy and reaches its highest level on the 21st day. It was found to be at the lowest level after birth.^57^ Factors that regulate COX-2 expression in cells and tissues can be specific for physiological processes and for the related tissues. For example, COX-2 expressed in granulosa cells can be induced by luteinizing hormone and follicle-stimulating hormone.^3^

Balaji et al.^58^ reported that COX-1 is the dominant isoform in the mouse seminal vesicle. The COX-2 isoform is expressed in the testis, and continuous inhibition of this isoform inhibits sperm maturation.^59^ The expression levels of COX-1 and COX-2 in sperm are increased under the conditions of varicocele and diabetes.^60^

### Heart and Platelets

The use of NSAIDs and celecoxib or rofecoxib increases risk for cardiovascular events in medical conditions such as diabetes mellitus, congestive heart failure, dyslipidemia, and colorectal adenomas. Additionally, at moderate doses, celecoxib was not found to be inferior to ibuprofen or naproxen in cardiovascular safety.^61^–^63^ Metabolites of COX play important roles in the homeostasis in an organism. The COX-1 isoform is involved in vascular homeostasis in platelets and in most tissues.^14^ The only isoform detectable in platelets is COX-1.^9^ The role of COX-1 in thromboxane synthesis in platelets is explained by their inability to produce an inducible enzyme in response to activating conditions in seedless platelets.^15^

One study demonstrated that prostacyclin in vascular cells is produced by COX-2 and COX-1 under both pathological and physiological conditions.^5^ Prostacyclin, a potent vasodilator, is one of the most important prostanoids that controls the homeostasis of the cardiovascular system. In addition, it inhibits leukocyte adhesion, platelet aggregation, and the proliferation of vascular smooth muscle cells.^4^

The function of COX-2 in the cardiovascular system remains largely unknown. Ischemic preconditioning has been shown to increase COX-2 expression and activity in the heart. This increase of COX-2 activity mediates protective effects against myocardial palpitation and myocardial infarction.^10^

### Brain

Although both isoforms have been found in the central nervous system, only the distribution of COX-1 has been reported in detail.^64^ The COX-2 isoform is continuously expressed in only a few organs, one of which is the brain.^9^ Cyclooxygenase-2 is found in neurons in the cortex and hippocampus of the brain but not in glia or vascular endothelial cells.^65^ Medium levels are found in the piriform cortex, pyramidal cells, neocortex, and amygdala. It is found at lower levels in the caudate–putamen, hypothalamus, thalamus, and striatum.^10^ The COX-2 isoform, expressed under normal conditions in the central nervous system, contributes to basic brain functions, such as memory consolidation, synaptic activity, and functional hyperemia.^66^,^67^

Cyclooxygenase-2 is highly expressed in the brain of neonates, indicating the importance of this isoform in modulating blood flow at this stage of life.^8^ Basal expression of COX-2 in developing and adult brain is regulated by natural synaptic activity.^65^

### Bone

Studies have shown that the inhibition or removal of COX-2 inhibits fracture healing, whereas the removal of COX-1 does not affect fracture healing. This indicates that the COX-2 isoform is primarily responsible for regulating fracture healing.^68^,^69^ The healing of tibial fractures was significantly delayed in COX-2^−/−^ mice compared to COX-1^−/−^ and wild-type controls.^70^ Fracture healing has been reported to be faster with the use of a retroviral vector to express the COX-2 isoform.^68^ These results indicate that COX-2 may be a necessary component in fracture healing and that agents modulating COX enzyme activity may directly affect bone repair.^71^

Since hemostatic and inflammatory processes are essential components of bone formation, COX enzymes play an important physiological role in fracture healing.^71^ Fracture healing is a complex three-stage process: (1) inflammation stage, (2) reparative stage, and (3) remodeling stage. The first stage begins immediately after fracture and is characterized by the formation of a hematoma, the migration of mesenchymal cells to the fracture site, and the release of growth factors and cytokines from fibroblasts and leukocytes. Following the initial inflammatory response, new bone is formed by intramembranous and endochondral ossification; these processes are mostly mediated by osteoblasts. These stages are followed by the long process of remodeling. Fracture healing is similar to many aspects of bone growth, but COX-2-mediated inflammation is an important initiation step in the fracture-healing process.^10^,^72^

### Inflammation

**Figure 4:**
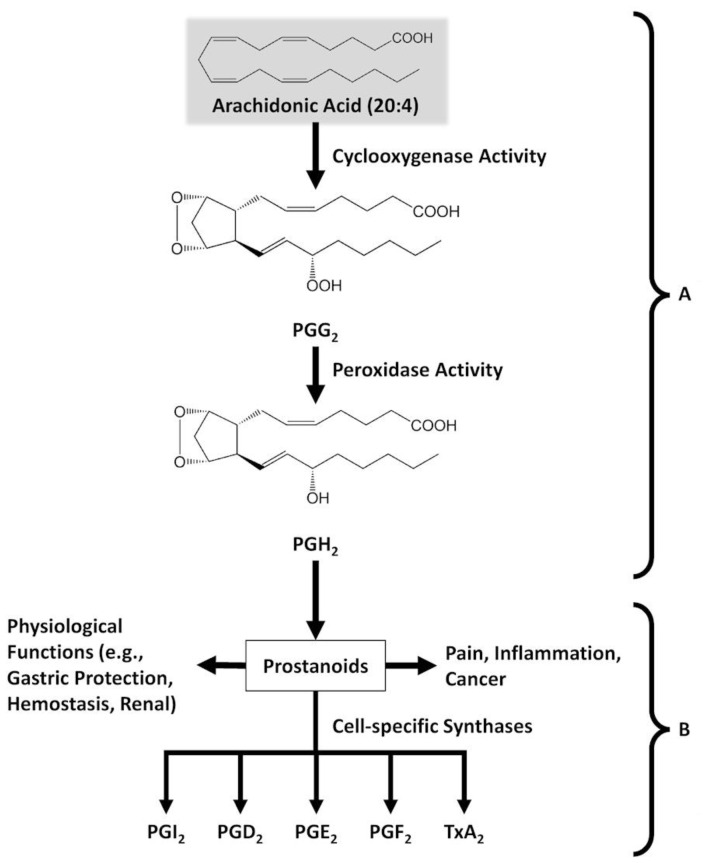
Biosynthesis and Main Biological Activities of Prostanoid Mediators from Arachidonic Acid. **A:** Cyclooxygenases catalyze two reactions: (1) cyclooxygenase activity, the conversion of arachidonic acid to prostaglandin (PG) G2; and (2) peroxidase activity, the conversion of PGG2 to PGH2. **B:** The cell-specific synthases that are involved in the conversion of PGH2 to the five principal prostaglandins (PGI2, PGD2, PGE2, PGF2α, TxA2), collectively named prostanoids. Prostacyclin (PGI2) protects the stomach mucosa from damage and prevents aggregation of blood platelets. PGD2 is a mediator in brain and mast cells. PGE2 is mostly involved in pain, fever, and inflammation. PGF2α is involved in labor and parturition. Thromboxane A2 (TxA2) is mainly found in blood platelets and promotes clotting of blood by inducing platelet aggregation.

The COX-2 isoform is reported to play a central role in the inflammation process, and inhibition of this isoform is sufficient to achieve the same therapeutic results as less specific inhibitors that also target the COX-1 isoform (Figure 4).^15^

Although the role of COX-2 is not fully understood, the relationship between its induction and nerve degeneration suggests that the COX-2 isoform may play a greater role in the selective loss of nerve connections.^8^ Central inhibition of the COX-2 isoform may be important in pain control.^10^ Prostaglandin E2 (PGE2) synthesis is induced by cytokines, which are released by the action of pyrogens such as lipopolysaccharide. Although COX-2 expression in the central nervous system is increased after lipopolysaccharide stimulation, the induction occurs in the endothelium of cranial blood vessels and in the microglia, not in neurons. Furthermore, it is clear that PGE2 in the febrile response is caused by the COX-2 isoform induced in endothelial cells of blood vessels perfusing hypothalamus in non-neuronal cells.^9^

In epithelial cells, COX-2 expression increases during inflammation.^15^ An increase in COX-2 levels as a result of an intrauterine infection was found to be associated with preterm delivery.^73^ The expression of COX-2 during wound healing contributes to the wound healing process. In animal wound models, COX-2 inhibition was shown to exacerbate inflammation and to delay wound healing.^15^

In joint inflammation models, COX-2 expression was increased but COX-1 expression remained unchanged.^74^,^75^ The role of COX-1 and COX-2 in chronic pain such as rheumatoid and osteoarthritis continues to be investigated.^10^

### Parkinson’s and Alzheimer’s Disease

The overexpression of COX-2 has been reported in neurodegenerative disorders.^26^ The COX-2 isoform increases brain parenchymal amyloid plaque formation, leading to Alzheimer’s disease. Relatedly, the inhibition of this isoform has been reported to reduce the risk of Alzheimer’s disease.^8^ However, Aisen et al.^76^ reported that low-dose naproxen or rofecoxib did not slow cognitive decline in mild–moderate Alzheimer’s disease.

### Cancer

Many studies have reported that COX enzymes play an important role in the development of tumors by affecting angiogenesis in tumor cells.^5^,^15^ The roles of the two isoforms in tumorigenesis are different. The COX-1 isoform is expressed in vascular endothelial cells and contributes to angiogenesis that is involved in tumor or endometrial growth, wound healing, and inflammation. Unlike the relatively small contribution of the COX-1 isoform to tumorigenesis, COX-2 is functional in tumor formation and tumor growth. The overexpression of COX-2 in tumor cells causes the cells to escape from apoptosis and invade the matrix.^5^

Many studies have shown that COX-2 is overexpressed in some pre-cancerous lesions and cancer types, including kidney, uterus, bowel, and bladder cancer.^14^,^36^,^77^ The contribution of COX-2 to the development of colon cancers supports a role in the control of cell growth that is apart from the COX-1 isoform.^3^ In human gastric adenocarcinoma tissues, COX-2 mRNA levels were increased compared to normal samples in gastric mucosal tissue, but COX-1 mRNA levels were not elevated in carcinoma.^9^ The prolonged increase of COX-2 expression following colitis increases the sensitivity to colon cancer.^28^

## CONCLUSION

The COX enzyme has two major isoforms that are structurally similar. The COX-1 isoform is physiologically expressed in almost all tissues and has a protective role. The COX-2 isoform is expressed physiologically in the uterus, brain, kidney, and during pregnancy at low levels, whereas in pathological conditions such as inflammation and cancer it is highly expressed, and this expression gradually increases. The inhibition of these isoforms has different consequences. Inhibition of COX-1 leads to gastroduodenal damage and renal dysfunction, whereas COX-2 inhibition results in anti-inflammatory/analgesic effects and limits cancer progression and renal dysfunction. Further studies are needed to more clearly define the roles of the known COX isoforms.

## Abbreviations

COX: cyclooxygenase
ER: endoplasmic reticulum
NSAIDs: non-steroidal anti-inflammatory drugs
PG: prostaglandin
